# Supplementary figures and images for: Species-Dependent Structural Variations in Single-Domain Antibodies
Source: Antibodies (Basel). 2025 Nov 25;14(4):100. doi: 10.3390/antib14040100 (PMC12729405; doi:10.3390/antib14040100)

Supplementary data

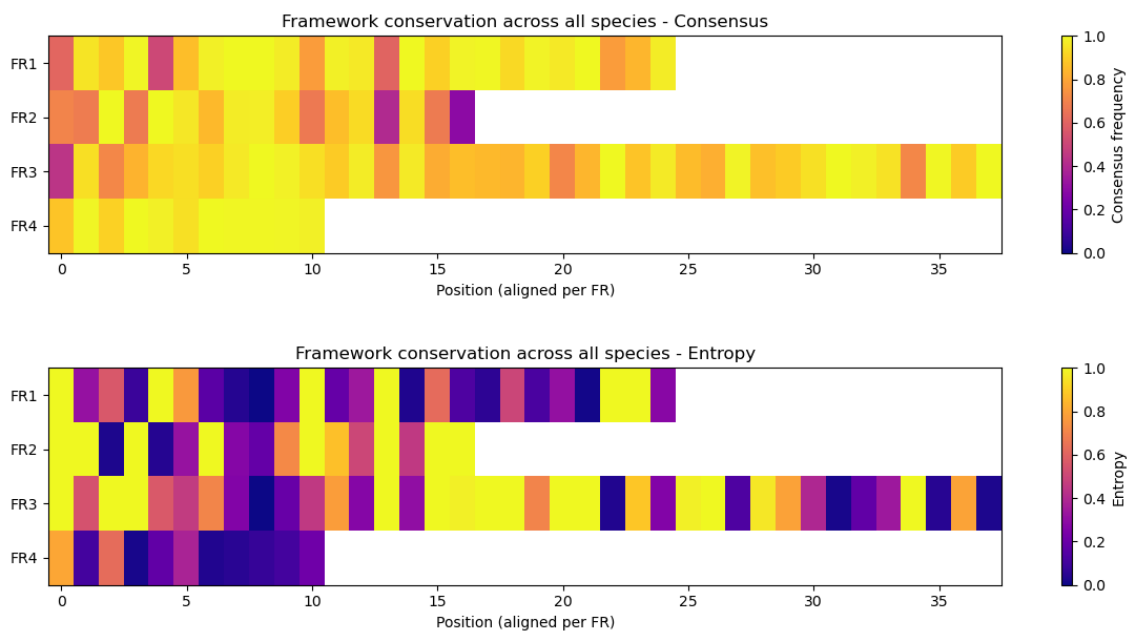

Figure S1. Conservation and entropy heatmaps across all species.

Supplement: Supplementary file 1 [file antibodies-14-00100-s001.zip › antibodies-3943711-supplementary.pdf]
